# Crystalline iron oxides stimulate methanogenic benzoate degradation in marine sediment-derived enrichment cultures

**DOI:** 10.1038/s41396-020-00824-7

**Published:** 2020-11-05

**Authors:** David A. Aromokeye, Oluwatobi E. Oni, Jan Tebben, Xiuran Yin, Tim Richter-Heitmann, Jenny Wendt, Rolf Nimzyk, Sten Littmann, Daniela Tienken, Ajinkya C. Kulkarni, Susann Henkel, Kai-Uwe Hinrichs, Marcus Elvert, Tilmann Harder, Sabine Kasten, Michael W. Friedrich

**Affiliations:** 1 Faculty of Biology/Chemistry, University of Bremen, Bremen, Germany; 2 MARUM—Center for Marine Environmental Sciences, University of Bremen, Bremen, Germany; 3 Alfred Wegener Institute, Helmholtz Centre for Polar and Marine Research, Bremerhaven, Germany; 4 Faculty of Geosciences, University of Bremen, Bremen, Germany; 5 Department of Biogeochemistry, Max Planck Institute for Marine Microbiology, Bremen, Germany

**Keywords:** Biogeochemistry, Microbial ecology

## Abstract

Elevated dissolved iron concentrations in the methanic zone are typical geochemical signatures of rapidly accumulating marine sediments. These sediments are often characterized by co-burial of iron oxides with recalcitrant aromatic organic matter of terrigenous origin. Thus far, iron oxides are predicted to either impede organic matter degradation, aiding its preservation, or identified to enhance organic carbon oxidation via direct electron transfer. Here, we investigated the effect of various iron oxide phases with differing crystallinity (magnetite, hematite, and lepidocrocite) during microbial degradation of the aromatic model compound benzoate in methanic sediments. In slurry incubations with magnetite or hematite, concurrent iron reduction, and methanogenesis were stimulated during accelerated benzoate degradation with methanogenesis as the dominant electron sink. In contrast, with lepidocrocite, benzoate degradation, and methanogenesis were inhibited. These observations were reproducible in sediment-free enrichments, even after five successive transfers. Genes involved in the complete degradation of benzoate were identified in multiple metagenome assembled genomes. Four previously unknown benzoate degraders of the genera *Thermincola* (Peptococcaceae, Firmicutes)*, Dethiobacter* (Syntrophomonadaceae, Firmicutes), Deltaproteobacteria bacteria SG8_13 (Desulfosarcinaceae, Deltaproteobacteria), and *Melioribacter* (Melioribacteraceae, Chlorobi) were identified from the marine sediment-derived enrichments. Scanning electron microscopy (SEM) and catalyzed reporter deposition fluorescence in situ hybridization (CARD-FISH) images showed the ability of microorganisms to colonize and concurrently reduce magnetite likely stimulated by the observed methanogenic benzoate degradation. These findings explain the possible contribution of organoclastic reduction of iron oxides to the elevated dissolved Fe^2+^ pool typically observed in methanic zones of rapidly accumulating coastal and continental margin sediments.

## Introduction

Microbial degradation of organic matter controls the carbon flux and biogeochemical cycling of elements in marine environments [1, 2]. In addition to organic matter of marine origin, sediments in high accumulation settings usually receive high amounts of terrigenous organic matter mainly composed of cellulose and lignin, a biopolymer rich in aromatic subunits [3–5]. Under conditions of high sediment deposition [6–13], poorly crystalline (e.g., lepidocrocite) and crystalline (e.g., magnetite and hematite) iron oxide phases are often co-buried with organic matter of terrigenous origin. These iron oxides play an important role in both organic carbon production and preservation in marine sediments [14, 15]. A substantial fraction of the global pool of organic carbon (21.5 ± 8.6%) is estimated to be bound to reactive iron phases in marine sediments [16]. This limits microbial organic carbon degradation by direct chelation or co-precipitation of macromolecular organic matter-iron structures [16].

The final mineralization steps in the degradation of organic matter are constrained by the reactivity of the organic matter (i.e., labile or recalcitrant) and availability of inorganic terminal electron acceptors (i.e., Fe/Mn oxides, sulfate, and CO_2_). In surface sediments, organic matter is generally labile, whereas the reactivity of organic matter decreases with increasing sediment depth and age [17, 18]. Thus, microorganisms inhabiting deeper sediments of high accumulation marine environments have to deal with recalcitrant organic matter bound to reactive iron oxide phases. This in turn limits their metabolic potential. There are indications for microbial iron reduction in the deep methanic zone highlighted by the detection of dissolved Fe^2+^ in pore water [6–13, 19]. Of the various microbial processes that could hypothetically fuel iron reduction in the deep methanic zone, iron oxide dependent anaerobic oxidation of methane (AOM) has been demonstrated [19]. Likewise, dissimilatory iron reduction is feasible but requires similar electron donors (acetate and H_2_) as methanogenesis [20, 21]. Another potential mechanism yet to be shown for marine sediments is fermentation-based organoclastic iron reduction. Fermentative iron reduction of poorly crystalline iron oxide phases accounts for up to 5% of electrons during organic matter degradation in pure culture studies [20, 22, 23]. On the other hand, crystalline iron oxide phases accelerate organic carbon degradation to methane as primary electron sink (i.e., methanogenic degradation) in rice field soils, lake, and marine sediments by serving as conduits for direct electron transfer [24–27]. Given these previous findings, a hitherto unexplored strategy for microbes to efficiently degrade recalcitrant organic matter may rely on crystalline iron oxides acting concurrently as electron acceptors while serving as conduits. Under such conditions, crystalline iron oxides would enhance both methanogenesis and degradation instead of preservation of recalcitrant organic matter while being partly reduced, which in turn releases Fe^2+^ into pore water. We therefore hypothesize that during the degradation of recalcitrant organic matter of terrestrial origin, crystalline portions of co-buried iron oxides act as conduits, thus facilitating efficient methanogenic organic matter degradation. The feasibility of this concurrent dual role of crystalline iron oxides has not yet been demonstrated in marine sediments and is the focus of this study.

Previously, we investigated the Helgoland Mud Area (HMA) in the North Sea, which is characterized by high accumulation of fine-grained sediments with elevated Fe^2+^ pore-water concentrations in the methanic zone [6, 19, 28]. We characterised the composition of the bio-available fraction of the organic matter utilized by the microbial communities therein [29]. It appears that aromatic compounds of terrestrial origin are preferentially degraded in the methanic zone of these deposits [29]. The findings from the HMA are consistent with recent findings indicating preferential degradation of humics-like substances during methanogenic degradation in sediments with high terrestrial organic matter loading [30]. Since benzoate is the central intermediate in the anaerobic degradation pathway of most aromatic compounds [31, 32], it is widely used as a model compound to study anaerobic degradation of aromatic hydrocarbons [31, 33]. Thermodynamic feasibility of methanogenic benzoate degradation under environmental conditions requires a complex syntrophic community of bacteria and archaea [34–36] according to the equation:

1 $$\normalsize 4{\mathrm{C}}_6{\mathrm{H}}_5{\mathrm{COO}}^ - + 4{\mathrm{H}}^ + \to 15{\mathrm{CH}}_4 + 13{\mathrm{CO}}_2;{\Delta}{\mathrm{G}}^{0\prime } \\ = - 624\;{\mathrm{kJ}}\;{\mathrm{per}}\;4\;{\mathrm{mol}}\;{\mathrm{benzoate}}$$

Given our previous findings, here, we enriched benzoate degrading microbial communities from the methanic zone of the HMA, and supplied these communities with iron oxides with distinct crystallinities. This was done in both sediment slurry incubations as well as over five successive sediment-free enrichments. By providing benzoate as the only added organic carbon source for these microbial communities, we evaluated the role that different iron oxides play during microbial degradation of recalcitrant organic carbon. Our results show (1) how the presence of crystalline iron oxides stimulates methanogenic benzoate degradation, (2) the possible dynamics of microbe-crystalline mineral interaction during recalcitrant organic matter degradation, and (3) novel benzoate degrading communities enriched from marine sediments.

## Materials and methods

### Benzoate degradation experiment within sediment slurry

Geochemical profiles and depositional history of sampling site, the HMA were previously described [6, 19, 28]. For the current study, sediments were taken during RV HEINCKE research expedition HE443 (54°05.23′N; 007°58.04′E) in May 2015 and preserved prior to incubation set up as described elsewhere [26]. In order to study benzoate degradation in the presence of iron oxides (lepidocrocite, hematite, and magnetite, Table S1), sediment samples from the methanic zone (247–279 cm) were used. Further details of the slurry preparation are presented in the Supplementary material.

### Cultivation of sediment-free benzoate degrading enrichment cultures

A modified strictly anaerobic sterile salt water enrichment medium was used for cultivation of the enrichment cultures following Widdel et al. [37]. The bicarbonate-buffered (30 mM) sulfate depleted basal medium contained 20 g/L NaCl, 3 g/L MgCl_2_·6H_2_0, 0.5 g/L KCl, 0.2 g/L KH_2_PO_4_, 0.25 g/L NH_4_Cl, and 0.15 g/L CaCl_2_·2H_2_O and 2 mM Na_2_S·9H_2_O as reducing agent. Trace elements, vitamin solution and selenite-tungsten were added respectively as previously described [37–39]. The pH of the complete medium was between 7 and 7.2 before dispensing into serum bottles and used for cultivation. Headspace of serum bottles were flushed and completely filled with N_2_–CO_2_ (80:20%). In the initial transfer from sediment slurry incubations, 2 mL of slurry from the sediment incubation from respective treatment types were transferred into salt water media described above and cultivated with 5 mM benzoate and 30 mM respective iron oxide (250 and 1500 µmoles respectively in 50 mL final volume). After methanogenesis was observed, these cultures served as first generation transfers. For subsequent transfers, 5 mL from the previous generation amounting to 10% of media volume was transferred. The cultivation media became completely sediment free after the second generation transfer. Continuous transfer was done until the fifth generation transfer where several triplicates of the benzoate-magnetite (BM5), benzoate (B5) and benzoate-lepidocrocite (BL5) treatments were made with similar concentration of benzoate and or respective iron oxides.

### Analytical methods

Methane concentrations in incubation headspace samples (100 µl) were monitored over time as previously described [26]. Because of the difficulty of getting an accurate determination of iron reduction kinetics by measuring total Fe(II) produced in sediment incubations [26], Fe^2+^ formation in aqueous phase of sediment incubations was monitored spectrophotometrically [40]. However in the sediment-free enrichment cultures, total Fe(II) was measured as described previously [26]. It was possible to accurately determine total Fe(II) because the provided iron oxide (magnetite or lepidocrocite) is the only iron oxide present in the enrichment medium unlike the slurry incubations.

To determine benzoate concentration in both the sediment incubations and sediment-free enrichment cultures, sterile deionised-water diluted 200 µl aliquot of supernatant (stored at −20 °C before analysis) obtained from centrifuged (20,000 × *g*) 1 ml sediment slurry was used to quantify benzoic acid by LC-MS. The LC-MS method is described in Supplementary material.

Intermediates from benzoate degradation (butyrate and acetate) were measured from deionised-water diluted aliquots (200 µL) from supernatant of the enrichment cultures following [41]. H_2_ measurements in the headspace were done at timepoints where methanogenesis was on-going following Lin et al. [42]. The results are not reported since H_2_ was undetectable.

### DNA extraction, 16S rRNA gene amplification, and sequencing

**Fig. 1:**
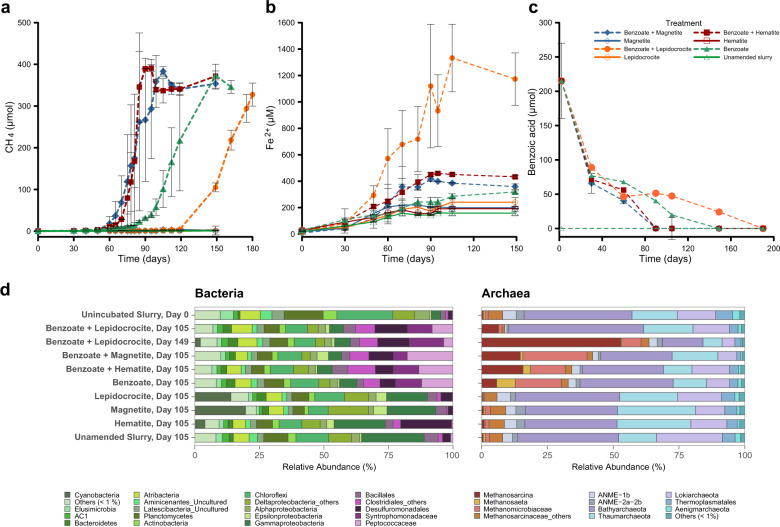
Concurrent methanogenesis and crystalline iron reduction during benzoate degradation in sediment slurry incubations. **a** Enhancement of methanogenesis by (semi)conductive crystalline iron minerals (hematite and magnetite) and inhibition of methanogenesis by nonconductive, poorly crystalline iron lepidocrocite, *n* = 3, error bars are 1 s.d. **b** Concurrent reduction of crystalline iron(III) minerals during phase of active methanogenesis (after day 60). Higher concentrations of dissolved Fe^2+^ were observed in the presence of hematite and magnetite as compared to controls with benzoate only. **c** Time course of benzoate degradation in incubations, obtained by measuring the decrease in benzoic acid concentrations. **d** Bacteria and archaea communities enriched during the sediment slurry incubations. Two types of color panels were used for the plots to differentiate communities stimulated during the incubation and presented on either order, family, or genus level from the communities whose relative abundance did not increase presented on phylum or class level.

DNA was extracted from 1 mL of slurry taken from incubation at certain timepoints using a direct lysis protocol [43] with modifications described elsewhere [26]. For the enrichment cultures, DNA was extracted from 6 ml of each culture; the 6 ml volume was dispensed into triplicate screw-cap tubes containing zirconium beads (0.1 mm diameter) and centrifuged (15,300 ×*g*, 5 min). Supernatant was removed from each tube, leaving behind ~200 µL followed by DNA extraction [26]. For sequencing, extracted biological replicate DNA from same treatment were pooled since CH_4_ and dissolved Fe^2+^ concentrations were similar in the treatments (Fig. 1a, b). In the sediment-free enrichment cultures, however, DNA extracts from each biological replicate were sequenced separately. PCR amplification, bacteria and archaea 16S rRNA Illumina HiSeq 4000 sequencing and subsequent analysis of sequencing methodology were described elsewhere [26].

### Metagenomic sequencing of highly enriched cultures

For metagenomic sequencing, 200 ng of extracted DNA from each of the three highly enriched cultures (BM5, BL5, and B5) were used as starting material. Each DNA sample was fragmented in a M220 Focused-Ultrasonicator (Covaris Inc., MA, USA) to an average fragment size of 550 bp according to the Illumina TrueSeq Nano DNA LT Library preparation protocol (Illumina Inc., San Diego, CA, USA). Sequencing run was performed on an Illumina MiSeq Sequencer using MiSeq V3 Reagent Kit according the manufacturer’s instructions. Analysis of resulting sequences was described in Supplementary material.

### SEM and CARD-FISH

After ~200 days of cultivating BM5, B5, and BL5 cultures, 500 µl aliquots of the triplicates from each of the three cultures were pooled together to evaluate the nature of microbe-mineral interactions in the enrichments. In total, 50 µl from each of the three pooled samples were fixed for SEM imaging using paraformaldehyde with a final concentration of 2% for 1 h at room temperature. The samples were rinsed 3× with PBS and some drops of the sample material were immobilized on silicon wafers (Ted Pella, USA). Sample materials were dehydrated with ethanol of different concentrations (30%, 50%, 80%, and 96% for 10 min). After dehydration, the samples were dried using a critical point dryer (EM CPD 300) (Leica, Wetzlar, Germany). The prepared samples were mounted on an aluminum stub with sticky carbon tape (PLANO GmbH, Wetzlar Germany). Mounted samples were imaged on a SEM (Quanta^TM^ 250 FEG, FEI Eindhoven, Netherlands) with either 2 kV (for secondary electron images) or 5 kV (back scattered electron images).

CARD-FISH was done on aliquots of the same sample material used for SEM. Horseradish peroxidase-labeled probes used were as follows: Arch 915 (general Archaea) and EUBI-III (general Eubacteria group I, II, and II as a mix, NON338 control probe (negative control)). All samples were washed 2 times with deionized water and dried on a 22 × 22 mm indium tin oxide coated glass slide (DELMIC, Netherlands). Density was checked under a stereo microscope to ensure appropriateness of cell material for FISH [44]. To stain bacteria/archaea DNA, slides were incubated in 1 ml Milli-Q water containing 1 µl of 1 µg/µl 4′,6-diamidin-2-phenylindole (DAPI) for 10 min at 4 °C. Slides were further washed in deionized water for 5 min and air dried. For fluorescence microscope imaging slides were embedded in a mix of Citifluor/Vectashield 4:1. The images were done with an Axio Imager M2 from Zeiss with ZEN-2 (blue version) software by 40× objective and 100× objective. The following channels were used to view the images: DAPI for bacteria/archaea DNA, FITC (EGFP-HC filter set, bacteria), Alexa594 (HC filter set TxRed, archaea), and bright field light (iron oxide).

## Results and discussion

Our study elucidates the dynamics of microbial interaction with crystalline iron oxides during organic matter degradation in methanic sediments from rapidly accumulating marine environments. By stimulating microbial communities involved in concurrent benzoate degradation, iron reduction and methanogenesis, the findings demonstrate how crystalline iron oxides enhance the ability of microorganisms to efficiently degrade recalcitrant organic matter in methanic marine sediments. The results presented also show sediment-free microbial communities involved in benzoate degradation with methanogenesis as primary electron sink. Finally, our study highlights four previously unidentified genera with capabilities to completely metabolize benzoate; *Therminicola*, *Dethiobacter*, *Melioribacter*, and Deltaproteobacteria bacterium SG8_13, based on their presence in the enrichments and genomic make-up.

### Concurrent iron reduction and methanogenesis in sediment incubations

As model iron oxides in our sediment incubations, we used hematite, magnetite, and lepidocrocite, which were selected based on their different degrees of crystallinity, conductivity and reactivity (Table S1) [45]. Substantial quantities of lepidocrocite were previously detected in the HMA sediments by Mössbauer spectroscopy [6]. In sediment slurry incubations with benzoate and (semi)crystalline iron oxides, i.e., hematite and magnetite, both methanogenesis and benzoate degradation were accelerated compared to the benzoate addition only and the lepidocrocite amended incubations. The accelerated methanogenic benzoate degradation in these crystalline iron-amended treatments was accompanied by a concurrent increase in dissolved Fe^2+^ concentrations (Fig. 1a–c). Both Fe^2+^ concentrations and methane amounts reached a plateau as benzoate concentrations dropped below the detection limit (90–95 days; Fig. 1a–c). In contrast, iron reduction was more pronounced in the incubations with benzoate and lepidocrocite amendments. Methanogenesis did not commence until no further increase in dissolved Fe^2+^ concentrations was observed (after 120 days; Fig. 1b). Iron reduction did occur in control incubations without addition of benzoate, but this was probably driven by oxidation of residual sedimentary organic matter. Dissolved Fe^2+^ concentrations in these controls were, however, not as high (e.g., 158 ± 15 µM in ‘Nothing Added’ incubation) as in the benzoate amended incubations (at least 318 ± 8 µM). Similarly, CH_4_ was not detected over time in the controls (Fig. 1a). Thus, the concurrent stimulation of crystalline iron reduction and methanogenesis in these sediment incubations was primarily driven by benzoate degradation. A positive correlation between methanogenesis and iron reduction both in situ and in incubation experiments was previously demonstrated during organic matter degradation in terrestrial environments. Examples include Arctic tundra and rice paddy soils that are rich in iron oxides and aromatic carbon compounds [46–48]. Similar processes may also explain the high Fe^2+^ concentrations in the methanic zone of sediments of the HMA and other marine or limnic environments [6–9].

Benzoate degraders under methanogenic conditions in marine sediments have not been identified to date. However, the bacteria communities stimulated by benzoate addition to the slurries (mostly families Peptococcaceae and Syntrophomonadaceae; Fig. 1d) were hitherto regarded as syntrophic benzoate degraders [27, 31, 49, 50]. The deltaproteobacterial order Desulfuromonadales harbors organisms known to perform microbial iron reduction in coastal marine sediments [26, 51]. Sequences falling into this order were found to increase in relative abundance from 1.5% at day 0 to between 6–12.6% at day 105 across all benzoate amended incubations (Fig. 1d). In addition to their role as iron oxide reducers, these microorganisms can potentially transfer electrons via mineral-mediated direct interspecies electron transfer (mDIET) to methanogens in the sediment matrix, thus resulting in accelerated methanogenesis in magnetite- and hematite-amended slurries [24, 26]. Therefore, the pathway of microbial utilization of iron oxides seems to involve both, reduction and use as conduits. Members of the genus *Methanosarcina, Methanosaeta*, and the family Methanomicrobiaceae were the dominant methanogens enriched during the phase of active methanogenesis (Fig. 1d). These enriched communities were specifically stimulated by benzoate addition only (Fig. 1d).

### **Concurrent iron reduction and methanogenesis in fifth generation sediment-free enrichmentcultures**

Given the observation of concurrent reduction of crystalline iron oxides and methanogenesis in the sediment slurry incubations, we cultivated the microbial communities over five successive transfers in an artificial enrichment medium. The aim was (I) to gain a better understanding of how electrons from benzoate degradation may fuel both iron reduction and methanogenesis without interference of the sediment matrix, and (II) to obtain active enrichment cultures performing benzoate degradation in marine sediments. For the fifth generation physiological experiments, ca. 250 µmol of benzoate was fed to each of the triplicate bottles of the benzoate-only enrichment (B5), benzoate-magnetite enrichment (BM5) representing crystalline iron oxides and benzoate-lepidocrocite enrichment (BL5) representing poorly crystalline iron oxides. We monitored methane formation, iron reduction (where possible), build-up and eventual depletion of fermentation intermediates during benzoate degradation.

**Fig. 2:**
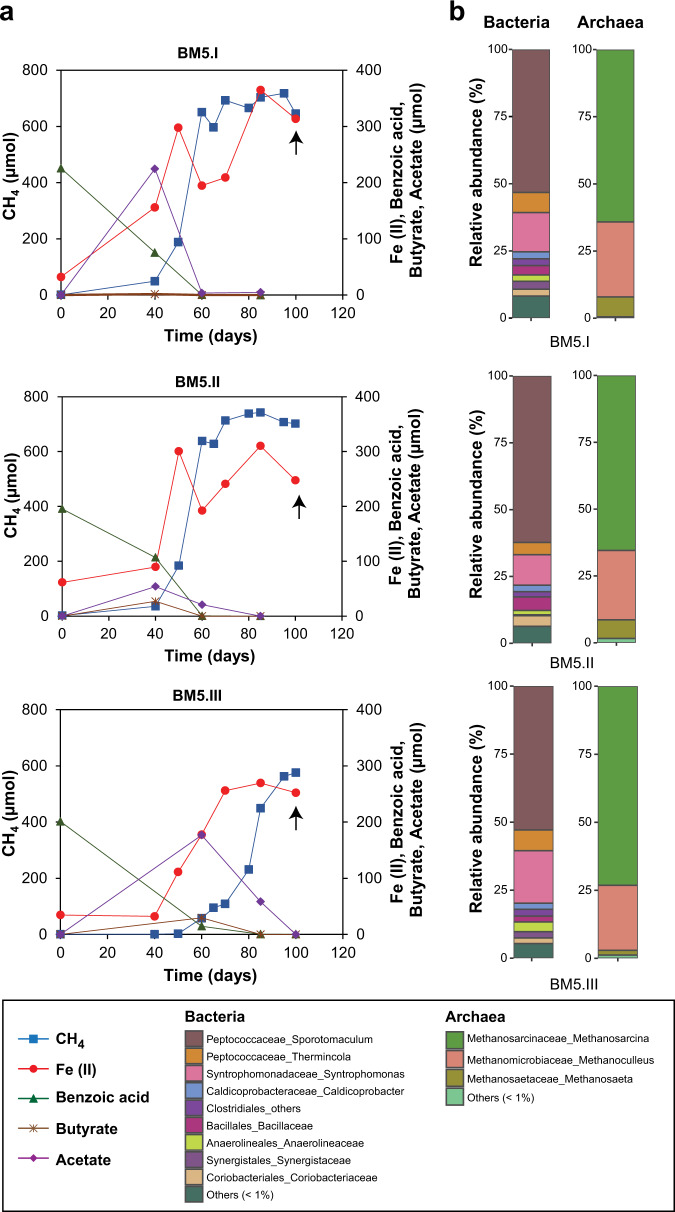
Concurrent iron reduction and methanogenesis in BM5 enrichment cultivated with benzoate (represented by its benzoic acid derivative) and magnetite as substrates in the  fifth generation. **a** Kinetics of benzoic acid degradation, build-up and removal of intermediates (butyrate and acetate), increasing Fe(II) and CH_4_ amounts over time. **b** 16S rRNA gene derived bacterial and archaeal community composition presented on family or genus level. Arrows in (**a**) reflect the time point from which DNA was extracted for sequencing.

**Fig. 3:**
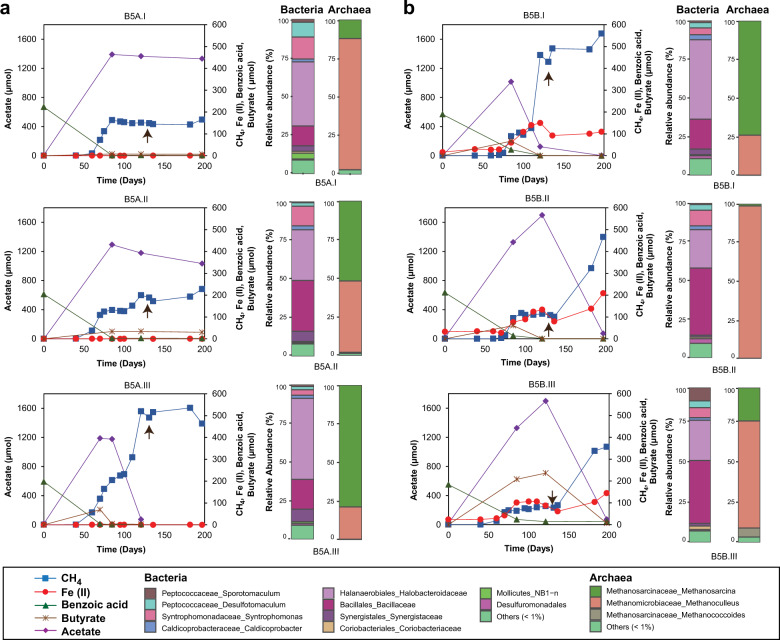
Kinetics of benzoate degradation and the 16S rRNA derived microbial community composition in B5 enrichment cultivated without iron oxides after 5 transfers. **a** Time course of intermediate (acetate and butyrate) build-up, methanogenesis, and microbial community composition in triplicates from the habitual cultivation. Acetate, first detected after 85 days, stayed in the system until 200 days in 2 of 3 replicates. Thus benzoate was not turned over completely to CH_4_. **b** Kinetics of separate B5 triplicates amended with magnetite demonstrating that acetate accumulation was effectively removed by concurrent magnetite reduction as methanogenesis progressed. Arrows in both (**a**) and (**b**) indicate time point (day 136) sequenced for microbial community composition.

**Fig. 4:**
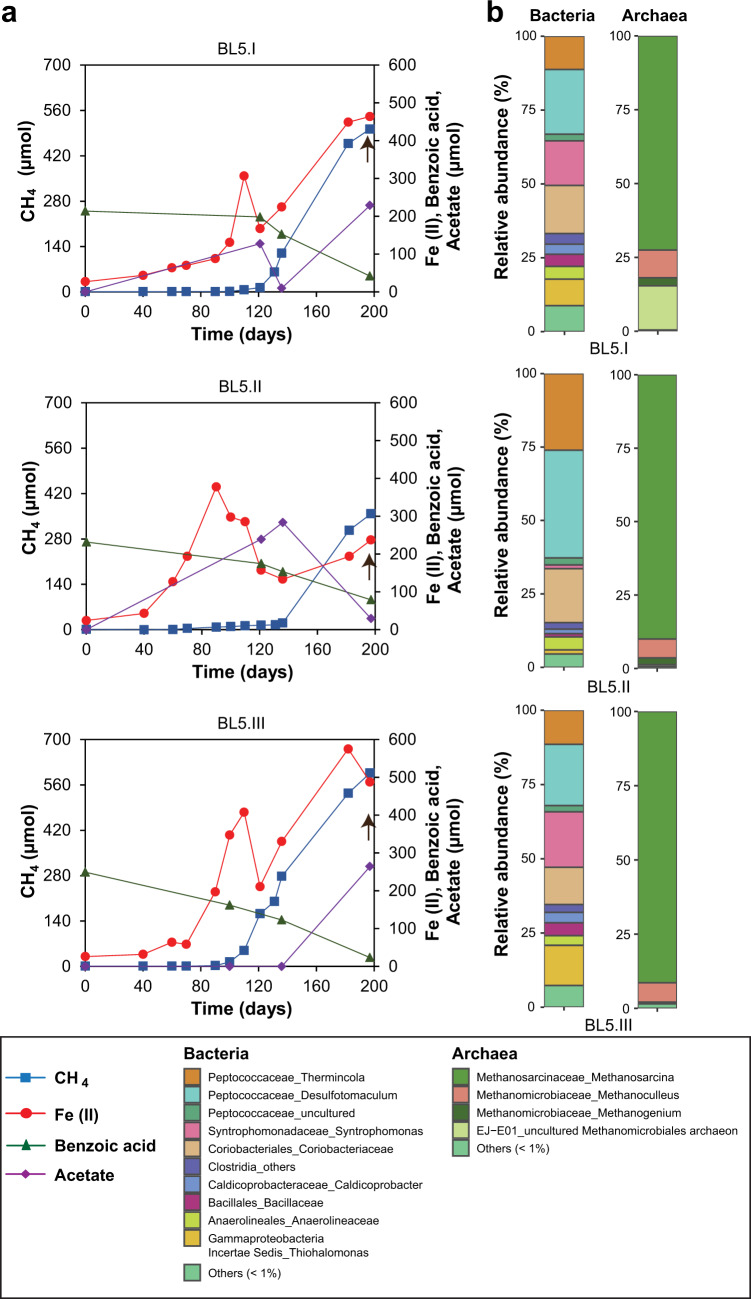
Kinetics of benzoate degradation and microbial community composition in BL5 enrichment (fifth generation) cultivated with lepidocrocite. **a** Time course of benzoate degradation, transient acetate build-up, iron reduction, and methanogenesis. **b** 16S rRNA gene derived microbial community composition after 197 days as indicated by arrows in (**a**).

In BM5, magnetite reduction occurred concurrently with methanogenic benzoate degradation (Fig. 2a) similarly to the benzoate-magnetite sediment incubation (Fig. 1). While H_2_ was not detected, a transient build-up of acetate (max. 200 µmol) and butyrate (max. 30 µmol) was measured. CH_4_ and Fe(II) amounts (see “Materials and methods”) plateaued after 100 days. For the B5 enrichment, methanogenic benzoate degradation was evident (Fig. 3a). However, methanogenesis appeared to be inhibited in two replicates (max. 200 µmol CH_4_ formed) after 121 days compared to the third replicate (520 µmol CH_4_). Instead, high amounts of acetate were measured in both replicates: 1290 and 1330 µmoles, even after 200 days. There was also a transient build-up of butyrate (up to 69 µmoles) over the 200 days. Compared to the BM5 (Fig. 2a) and B5 (Fig. 3a), the BL5 enrichment was characterized by slower and incomplete benzoate degradation after 200 days (Fig. 4a). Substantial amounts of benzoate (29–79 µmol) was still remaining in BL5 after 197 days, compared to only 3 µmol found in B5 (day 121) and absence of benzoate in BM5 (day 100). While butyrate was not detected in the timepoints sampled; acetate (up to 280 µmol) was still detected after 200 days showing that methanogenic benzoate degradation was limited in the presence of lepidocrocite. Therefore, while crystalline iron oxides stimulate methanogenic benzoate degradation, poorly crystalline lepidocrocite inhibits this process (Figs. 1–4). Inhibitory effects of lepidocrocite on benzoate degradation support previous findings that reactive iron oxide phases rather hamper microbial organic matter degradation [16, 52]. In general, iron reduction was more pronounced with lepidocrocite (Figs. 1b, 4). Enhanced iron reduction with the more reactive lepidocrocite likely resulted in the inhibition of methanogenesis; a phenomenon previously demonstrated when ferrihydrite was provided to ferruginous lake sediments incubations [53]. However, unlike the sediment incubation with lepidocrocite where iron reduction reached a plateau before methanogenesis onset (Fig. 1), concurrent methanogenesis and iron reduction was observed in the BL5 enrichment when methanogenesis started (Fig. 4a).

Microbial community compositions in the three enrichments were less diverse compared to the initial sediment incubations because the original sediment community was likely outcompeted in the enrichments (Figs. 1d, 2b, 3a, 4b). The communities in each enrichment also differed from one another in the all transfers (Figs. 2b, 3, 4b, and S1–S3). For example, *Sporotomaculum* (53–62%) dominated the BM5 enrichment, while Clostridia family Halobacteroidaceae (33–52%) was dominant in B5 and *Desulfotomaculum* (20–37%) in the BL5 enrichment. The genus *Syntrophomonas* was present in all enrichments (11–19% in BM5; 14–40% in B5; 2–18% in BL5). Some taxa such as *Thermincola* (5–8% in BM5; 11–26% in BL5) and Coriobacteriaceae (2–4% in BM5; 13–18% in BL5) were enriched in the presence of iron oxides. Some genera were particularly enriched in individual enrichments such as *Caldicoprobacter* (2%) in BM5, the order Bacillales (13–33%) in B5 and *Thiohalomonas* (2–14%) in BL5. In general, most of the enriched genera described above for the enrichment cultures and in the initial sediment incubations (Fig. 1d) were members of the phylum Firmicutes. This emphasizes the central role of Firmicutes as key benzoate degraders in our study from marine environment, as previously shown for terrestrial environments [27, 31, 49, 50]. The archaea community was less diverse, comprising mostly *Methanosarcina* (64–73% in BM5; 15–80% in B5; 73–92% in BL5) *Methanoculleus* (24–28% in BM5; 7–9% in BL5 and below 1% in B5), and *Methanosaeta* (2–7% in BM5; below 1% in B5 and BL5).

The potential for concurrent crystalline iron oxide reduction and methanogenesis was also investigated. Another triplicate setup was prepared at the start of the fifth transfer and amended with 1500 µmoles magnetite as with the BM5 enrichment. Similarly to the BM5, the enrichment B5 could also concurrently perform magnetite reduction and methanogenesis, albeit at slower rates (Figs. 2a, 3b). Transient build-up of acetate was not as high (up to 230 µmoles) as in the B5 enrichment without magnetite amendment (up to 1330 µmoles) likely because electrons could be transferred more quickly to methane via magnetite. Inhibitory effects of acetate on syntrophic benzoate degradation either by adding a thermodynamic barrier or by affecting the proton electrochemical gradient was previously demonstrated [34, 36, 54]. Under such conditions, thermodynamic barriers placed by acetate accumulation were removed by providing the co-cultures with an acetate oxidizing syntroph [36]. Here, we showed that the presence of magnetite could remove such thermodynamic barrier caused by acetate accumulation [54–56]. The microbial community composition after 197 days was similar to the habitual B5 enrichment. Therefore the same stable microbial community, enriched over four generation of successive transfers without iron oxides, which struggled to completely convert acetate to methane, could as well reduce magnetite and produce more methane concurrently, when provided with magnetite (Fig. 3).

Methanogenesis, when feasible, was the dominant electron sink in both, the sediment incubations and in the enrichment cultures (Tables 1 and 2). Both acetate and butyrate as intermediates of benzoate degradation were also completely degraded in the enrichment when methanogenesis was favored. The formation of butyrate as intermediate is particularly interesting as it points to a secondary fermentation pathway that converts butyrate to acetate and/or H_2._ Methanogenic benzoate degradation and the complete disappearance of intermediates from the enrichment were fastest in the BM5 culture enriched with magnetite (Fig. 2). Removal of the high amounts of acetate in the B5 enrichment, when provided with magnetite (Fig. 3b), further illuminated the beneficial role of crystalline iron oxides to stimulate organic matter degradation in the environment. The lack of increased Fe(II) amounts in controls without microbial cells (Fig. S4) also supports the conclusion that iron reduction in the enrichments is driven by microbial activity.

**Table 1:** Electron balance for benzoate degradation in highly enriched cultures forming CH_4_ and Fe(II). Addition of 5 mM benzoate as carbon substrate amounted to 250 µmol benzoate. The electron balance for methane formation was calculated using the expected stoichiometry for methanogenic benzoate degradation to methane and CO_2_ (see Eq. (1)). For iron(III) reduction, a stoichiometry of 30 Fe(II) ions formed per molecule of benzoate fully oxidized to 7 CO_2_ was assumed.

Enrichment	CH_4_ formed (µmol)	Electrons recovered in CH_4_ formed (%)	Fe(II) formed (µmol)	Electrons recovered in Fe(II) formed (%)	Total electron balance for benzoate turnover (%)
BM5	717.6	76.5	340	4.5	81.1
BM5	742.7	79.2	285.5	3.8	83.0
BM5	575.9	61.4	245	3.3	64.7
B5A.1	165.7	17.7	NA	NA	NA
B5A.2	227.9	24.3	NA	NA	NA
B5A.3	535.4	57.1	NA	NA	NA
B5B.1	559.3	59.7	125	1.7	61.3
B5B.2	466.7	49.8	184	2.5	52.2
B5B.3	356.7	38.	119.5	1.6	39.6
BL5	502.4	53.6	454	6.1	59.6
BL5	357.6	38.2	368	4.9	43.1
BL5	596.5	63.6	565	7.5	71.2

*B5A* habitual cultivation of the benzoate-only enrichment without iron oxides, *B5B* amendment of separate B5 enrichment replicates with magnetite, *NA* not applicable.

**Table 2:** Electron balance for methanogenic benzoate degradation in original sediment incubations. Addition of 5 mM benzoate as carbon substrate amounted to 187.5 µmol benzoate. Electron balance for methanogenesis calculated as for Table 1.

Treatment	CH_4_ formed (µmol)	Electrons recovered in CH_4_ formed from benzoate degraded (%)
Benzoate + Hematite	389.5	55.4
Benzoate + Magnetite	383.2	54.5
Benzoate only	353.6	50.3
Benzoate + Lepidocrocite	327.6	46.6

### Marine sediment-derived microbial communities involved in benzoate degradation

**Fig. 5:**
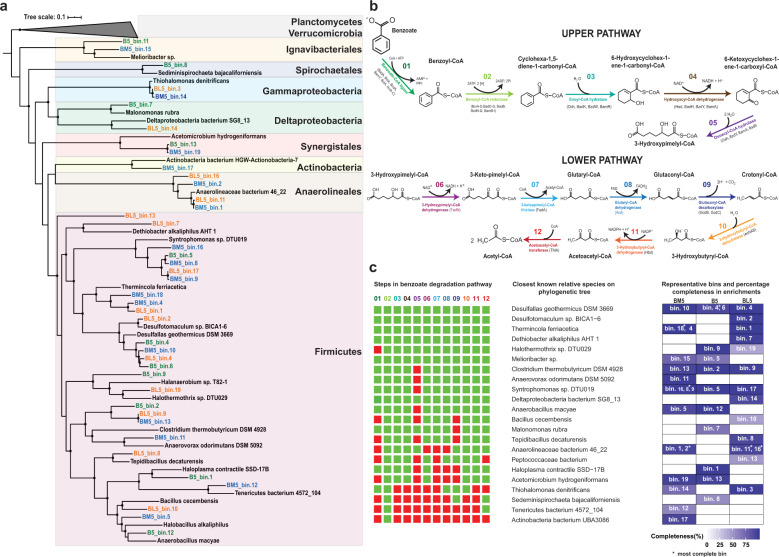
Evaluation of benzoate degradation potential in multiple MAGs from the highly enriched BM5, B5, and BL5 cultures. **a** Maximum likelihood phylogenomic tree showing closest bacterial relatives of MAGs obtained in this study. **b** Pathway and genes involved in complete degradation of benzoate to acetate. Genes catalysing the different steps of the benzoate degradation pathways are displayed in different colors for each of the 12 steps in the pathway. **c** Evaluation of the presence of genes required for complete benzoate degradation and level of completeness of all MAGs retrieved from all enrichment cultures. The color codes for each step in the pathway represent the various genes described in (**b**). To show presence of a particular gene, the boxes are marked green while the absence of a gene in the obtained MAGs was reflected with red boxes. The intermediate formation of pimelyl-CoA and its subsequent conversion to 3-hydroxypimelyl-CoA was ignored in scheme presented (**b**) since it was only previously shown for phototrophic α-Proteobacteria *Rhodopseudomonas palustris* [58]. Eventually *R. palustris* converts pimelyl-CoA to 3-hydroxypimelyl-CoA like other known benzoate degraders. Quality metrics of the MAGs are available in Tables S2–S5.

Multiple nearly complete metagenome assembled genomes (MAGs) containing genes involved in the various steps during anaerobic benzoate degradation were recovered from each of the three highly enriched cultures (Fig. 5a). Each of these MAGs contained genes involved in all steps, most steps, or few steps of the well-studied pathway of anaerobic benzoate degradation (Fig. 5b) [31]. The upper degradation pathway in Fig. 5b involves benzoate activation to benzoyl-CoA, de-aromatization of benzoyl-CoA, and β-oxidation leading to the formation of 3-hydroxypimelyl-CoA. The majority of the genes involved in the upper degradation pathway (step 1–5; Fig. 5b, c) were found in 32 of the 41 obtained MAGs in all three enrichments. Genes required for pathway utilized to convert 3-hydroxypimelyl-CoA to acetate (lower pathway, Fig. 5b) were more complete in the obtained MAGs compared to genes involved in the upper pathway (Fig. 5b, c). Specifically, the ATP-dependent benzoate-CoA ligase gene for benzoate activation to benzoyl-CoA was found in multiple MAGs in the three enrichments (Fig. S5). Some of these MAGs affiliated to the genera *Melioribacter*, *Haloplasma*, *Malonomonas*, and D. bacterium SG8_13 contained deeply branching benzoate-CoA ligase (Fig. S5), indicating the presence of novel benzoate-CoA ligase genes. Based on the presence of all genes required for complete benzoate degradation, we identified specialist microorganisms that completely metabolize benzoate to acetate. Most of these specialist MAGs belonged to the phylum Firmicutes (e.g., the genera *Desulfotomaculum*, *Thermincola*, *Dethiobacter*). Other identified specialist MAGs were affiliated to *Melioribacter* (phylum Ignavibacteria) and D. bacterium SG8_13 (family Desulfosarcinaceae, phylum Proteobacteria*)* (Fig. 5c).

The genus *Desulfotomaculum* is a heterogeneous group of spore-formers with high phylogenetic divergence and has been recently classified into 6 subclusters Ia-If [57]. *Desulfallas* and *Sporotomaculum* belong to the *Desulfotomaculum* subcluster Ib. Thus, while the 16S rRNA sequences classified the dominant groups in the incubations as *Sporotomaculum* (Figs. 2–4), the four MAGs obtained were more closely related to *Desulfallas geothermicus* based on the created genome tree (Fig. 5a). While *Desulfotomaculum* spp. have been previously identified as benzoate degraders in other environments [33, 49, 58, 59], *Thermincola, Dethiobacter*, and *Melioribacter* have not yet been shown to metabolize benzoate. *Melioribacter sp*. belongs to the recently proposed Ignavibacteriae phylum [60]. Representative *Melioribacter* species, i.e., *M. roseus*, are facultative anaerobes capable of fermentation of complex organic substrates and iron reduction [60, 61]. Here, we show that *Melioribacter* found in the BM5 and B5 enrichments can completely metabolize benzoate and possibly reduce magnetite. However, *Melioribacter* made up less than 1% of the relative abundance of the total bacterial community composition based on 16S rRNA gene analysis (see Figs. 2, 3). *Thermincola* was highly enriched with magnetite and lepidocrocite in all successive transfers (Figs. 2 and 4, and S1, S3). Two *Thermincola* species (*T. ferriacetica* and *T. potens*) have been shown to be capable of iron reduction with acetate as electron donor [62–64]. While *T. ferriacetica* has not been tested for growth on benzoate yet, *T. potens* failed to grow on benzoate in a microbial fuel cell system [65]. Here, we show in three different nearly complete MAGs closely related to *T. ferriacetica* that *Thermincola* likely concomitantly metabolized benzoate while reducing either magnetite or lepidocrocite in our enrichments (Fig. 5c). *Dethiobacter* belongs to the well characterized family Syntrophomonadaceae known for their ability to participate in syntrophic benzoate degradation [49]. They have been previously shown to use short chain fatty acids and H_2_ as electron donors with sulfur compounds as electron acceptors [66, 67]. The *Dethiobacter* MAG obtained from our BL5 enrichment contained all genes required for benzoate degradation, therefore expanding the metabolic capabilities of this relatively understudied Syntrophomonadaceae genus. Some sulfate reducers from the families Desulfobulbaceae, Desulfobacteriaceae, Desulfomicrobiaceae, and Syntrophobacteraceae were recently identified in a river sediment enrichment stimulated to couple benzoate degradation to sulfate reduction [68]. However, sulfate reducers can hardly completely metabolize benzoate to acetate [69] with the exception of *Desulfoprunum benzoelyticum* (family Desulfobulbaceae) [70]. Here, a MAG with genes for complete benzoate degradation from a known sulfate reducing family (Desulfosarcinaceae) closely related to Deltaproteobacterium bacteria SG8_13 was identified in the BL5 enrichment.

We found other members of the microbial community in the enrichments that lacked one or more genes needed to completely metabolize benzoate (Fig. 5c). Among this group, *Clostridium*, *Anaerovorax*, *Syntrophomonas, Halothermothrix*, and *Malonomonas* stood out as they lacked just one gene to facilitate complete benzoate degradation based on the data we obtained (Fig. 5c). However the absence of a gene in these MAGs might be due to incompleteness of the metagenome assembly. Particularly for the *Halothermothrix* MAG in the B5 enrichment, although 98.5% complete, the benzoate-CoA ligase for benzoate activation was missing. The predominance of this genus represented on family level as Halobacteroidaceae in the fifth transfer (52% relative abundance of bacteria 16S rRNA genes, Fig. 3) and its high enrichment since the second transfer (Fig. S3) suggests it is likely capable of complete benzoate degradation. Halobacteroidaceae, who are strictly anaerobic, gram negative fermentative halophiles [71] have not been previously linked to benzoate degradation.

Benzoate fermentation to butyrate via crotonyl-CoA and the subsequent secondary fermentation of butyrate is well established for members the family Syntrophomonadaceae [50, 72]. The likelihood of a secondary fermentation of the detected intermediate butyrate (Figs. 2 and 3) in addition to direct benzoate fermentation to acetate was reflected in the microbial community composition given the enrichment of *Syntrophomonas* spp. (Figs. 2–5). They were highly enriched based on 16S rRNA genes sequencing (Figs. 2b, 3b, 4b) and MAGs closely related to *Syntrophomonas sp*. DTU019 were found in all three enrichments (Fig. 5a, c). The ability of co-existing syntrophic degraders of aromatic compounds to utilize diverse alternative metabolic pathways increases overall thermodynamic favorability and decreases thermodynamic sensitivity to H_2_ (i.e., via H_2_ production) [73]. This might explain why H_2_ was not detected as an intermediate in our enrichments, which is corroborated by the transient accumulation of acetate and butyrate. Most of the MAGs lacking the benzoate-CoA ligase genes (Fig. 5c) needed for benzoate activation are putative candidates for syntrophic degradation of benzoate, taking up fermentation intermediates and participating in the lower steps of the benzoate degradation pathway.

Previously identified dissimilatory iron reducers in marine sediments from the family Desulfuromonadaceae [51] were not enriched in these cultures, despite the high amount of Fe(II) generated. Instead, the only known iron reducer that was predominantly found in our enrichments was *Thermincola*: 5–8% in BM5 (Fig. 2), 11–26% in BL5 (Fig. 4) and absent in the noniron-amended B5 enrichment. Therefore, we suggest that iron reduction in these cultures is not entirely dissimilatory but also tightly linked to benzoate degradation (fermentative iron reduction). Actinobacterial bacteria from the family Coriobacteriaceae which was previously enriched with iron oxides [74], were enriched in BM5 (2–4%). The actinobacterial MAG obtained from the BM5 enrichment lacked nearly all genes required for benzoate degradation. Thus, Coriobacteriaceae must have served solely as magnetite reducers in BM5 with the intermediate acetate as electron donor.

### Imaging reveals microbe-mineral interaction in highly enriched cultures

**Fig. 6:**
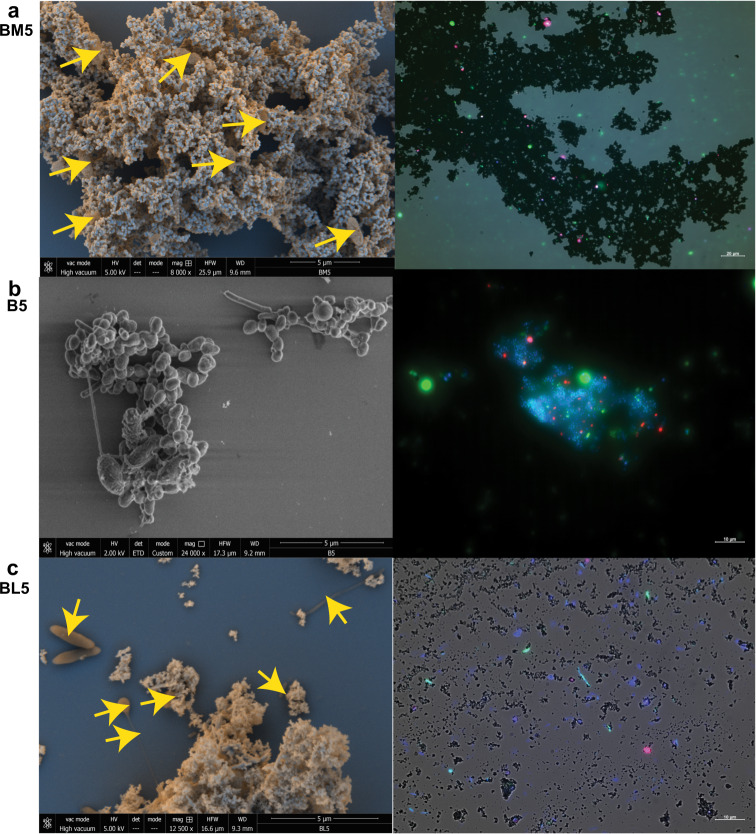
SEM and CARD-FISH images from highly enriched fifth generation cultures after 200 days. **a** BM5 enrichment on magnetite. **b** B5 enrichment cultivated without iron oxides. **c** BL5 enrichment cultivated with lepidocrocite. Left panels show the SEM images while right panels show CARD-FISH images. Yellow arrows in the left panels point to microbial cells. SEM images in (**a**) and (**c**) combined images from back scattered (blue colour) and secondary electron (orange colour) to clearly distinguish between microbial cells and iron oxides.

SEM and CARD-FISH imaging were performed after 200 days of incubating the fifth generation enrichment. The aim was to identify patterns of microbial interaction with either crystalline magnetite (BM5) or poorly crystalline lepidocrocite (BL5) in comparison to the control enrichment without iron oxides (B5). The expectation was that enriched microorganisms interact with either magnetite or lepidocrocite during benzoate degradation, which in turn enhances or limits methanogenic benzoate degradation. In the BM5 enrichment, microbial cells were observed on all magnetite particle surfaces by both SEM and CARD-FISH (Figs. 6a, S6). In contrast, for the B5 control enrichment, closely knitted dense cell aggregates were formed in the absence of a crystalline iron oxide surface (Figs. 6b, S7). This reflects the conducting role of magnetite as electrons could be transported between benzoate degrading bacteria and methanogenic archaea over long distances along the magnetite surface. Meanwhile, in the control B5 incubations, closer cell–cell distances might be advantageous for interspecies electron transfer as fermentation intermediates may be lost with longer cell–cell distances. As the Fe(II) measurements showed (Fig. 2), partial reduction of magnetite occurred; reflecting the availability for this less reactive iron oxide to serve as electron acceptor as well. A recent study used SEM to demonstrate that *Geobacter* can partly reduce an iron oxide mineral while colonizing its surface, with the partial reduction attributed to *Geobacter* being able to form direct cell–mineral contact [75]. Similarly acetoclastic methanogenesis was enhanced in *Methanosarcina mazei* when provided with magnetite nanoparticles while magnetite nanoparticles colonized the methanogen cell [76]. This type of cell–mineral contact with a conductive mineral like magnetite likely conferred the ability to stimulate methanogenic benzoate degradation while magnetite was also partly dissolved.

For BL5, the pattern of microbial cell distribution around reactive lepidocrocite was markedly different compared to BM5 grown on magnetite (Figs. 6c, S8). A mixture of single and aggregated cells was observed. Cell agglomerations were also sparser in BL5 unlike in BM5 and B5 enrichments. Both SEM and CARD-FISH images reflected that lepidocrocite was mostly dissolved, and the lepidocrocite enrichment lacked the typical cell aggregates seen with magnetite. This is possibly due to the poor crystallinity of nonconductive lepidocrocite. Thus, syntrophic communities growing with lepidocrocite mostly established longer cell-lepidocrocite distances, and did not form dense aggregates as seen in the control (B5, Fig. 6b). The inability to form dense cell aggregates and interaction with a nonconductive iron oxide likely resulted in the slower rates of benzoate degradation in the BL5 enrichment compared to B5 and BM5 respectively.

### Fermentative iron reduction contributes to Fe^2+^ pool in iron oxide-rich methanic marine sediments

**Fig. 7:**
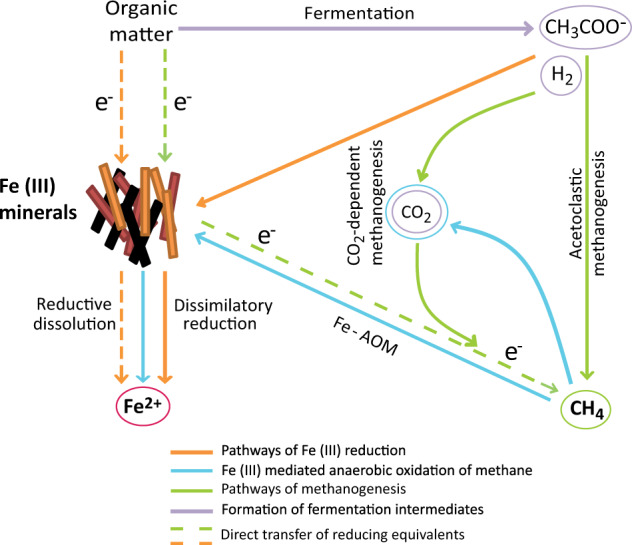
Schematic description of the various biotic processes that possibly contribute to the Fe^2+^ pool detected in pore water of iron oxide-rich methanic marine sediments, e.g., Helgoland Mud Area. This study presents an additional perspective and argues for the involvement of organoclastic iron reduction, in addition to Fe-AOM, as mechanism fueling methanic zone iron reduction. Red, brown and black colorations are representative of the various forms of Fe(III) present in the environment.

This study presents novel results explaining the role of different iron oxide phases to either inhibit or enhance organic matter degradation, and novel bacteria community, dominated by members of the phylum Firmicutes, involved in benzoate degradation in marine sediments. In terms of relevance to the environment, our observations, especially in the slurry incubations, demonstrate how both iron reduction and methanogenesis concurrently profit from crystalline iron oxide stimulated organic matter degradation. Such interaction between active microorganisms, iron oxides, and organic matter of rapidly accumulating marine sediments might be the source of the widely detected Fe^2+^ in pore water in the methanic zone. The physiological data presented in our sediment incubation and enrichment experiments are supported by a previous geochemical modeling study with Baltic Sea methanic sediments. Therein, it was proposed that the level of crystallinity and conductivity of iron oxides play a key role in determining whether methanogenesis is stimulated or constrained in iron oxide-rich marine environments [7]. The crystalline iron mineral phases may also be partly reduced in the process of mineral-mediated electron transfer between fermentative bacteria and methanogens as shown in Figs. 1–4. Thus, our study provides evidence for organoclastic iron reduction in addition to Fe-AOM [19] as mechanism driving microbial iron reduction in methanic marine sediments as shown in the scheme presented in Fig. 7. We argue that some of the Fe^2+^ detected in pore water of methanic subsurface sediments like the HMA originates from organoclastic iron oxide reduction during methanogenic fermentation of complex organic matter. The possibility of co-occurring Fe-AOM and iron reduction-linked methanogenic degradation of organic matter may therefore explain the observed correlation between organic matter degrading bacteria (e.g., Atribacteria [77, 78] and Burkholderiales [79]), and methanogenic/methane oxidizing archaea with high Fe^2+^ concentrations in some methane-rich sub-seafloor environments [6, 79]. The individual contributions of fermentative microorganisms [80], dissimilatory iron reducers, and methanogens [81, 82] to iron reduction remain unclear. Further studies are therefore required to better understand (I) the mechanistic details of such interdependencies between microbial cycling of organic matter and iron, and (II) how the less reactive iron oxides support microbial life in the deep biosphere representing the biggest reservoir of organic carbon on Earth [83].

## Supplementary information

Supplementary Materials and Results

## Data Availability

Raw sequence data used in this study can be accessed from GenBank Short Reads Archive with bioproject accession number PRJNA630030.
