# Supplementary material for: Crystalline iron oxides stimulate methanogenic benzoate degradation in marine sediment-derived enrichment cultures
Source: ISME J. 2020 Nov 5;15(4):965–80. doi: 10.1038/s41396-020-00824-7 (PMC8115662; doi:10.1038/s41396-020-00824-7)
Supplement: Supplementary file 1 — Supplementary Materials and Results [file 41396_2020_824_MOESM1_ESM.pdf]

Supplementary material

Crystalline iron oxides stimulate methanogenic benzoate degradation in marine sediment- derived enrichment cultures

David A. Aromokeye[1,2], Oluwatobi E. Oni[1], Jan Tebben[3], Xiuran Yin[1,2], Tim Richter-Heitmann[1], Jenny Wendt[2,4], Rolf Nimzyk[1], Sten Littmann[5], Daniela Tienken[5], Ajinkya Kulkarni[1], Susann Henkel[2,3], Kai-Uwe Hinrichs[2,4], Marcus Elvert[2,4], Tilmann Harder[1,3], Sabine Kasten[2,3,4] and Michael W. Friedrich[1,2*]

[1]Faculty of Biology/Chemistry, University of Bremen, Bremen, Germany

[2]MARUM – Center for Marine Environmental Sciences, University of Bremen, Bremen, Germany

[3]Alfred Wegener Institute, Helmholtz Centre for Polar and Marine Research, Bremerhaven, Germany

[4]Faculty of Geosciences, University of Bremen, Bremen, Germany

[5]Department of Biogeochemistry, Max Planck Institute for Marine Microbiology, Bremen, Germany

Running title: Iron oxides stimulate methanogenic benzoate degradation

* Corresponding author:

Correspondence:

Michael W. Friedrich,

Microbial Ecophysiology Group, Faculty of Biology/Chemistry, University of Bremen, PO Box 33 04 40, D-28334 Bremen, Germany

Email: michael.friedrich@uni-bremen.de

Supplementary methods

Slurry preparation

Several anoxic slurry incubations (1:3 w/v) were made in 120-mL serum vials with sulfate-free artificial sea water (ASW; composition [L^{-1}]: 26.4 g NaCl, 11.2 g $MgCl_2$, 1.5 g $CaCl_2.2H_2O$ and 0.7 g KCl). To triplicate vials, 5mM sodium benzoate and 30 mM iron oxides (lepidocrocite, hematite or magnetite; LanXess GmbH, Germany) were added. In addition, slurry incubations ($n=3$) devoid of either sodium benzoate or the aforementioned iron oxides (30 mM) were prepared to serve as background controls. Since samples were taken from depths of active methanogenesis (1) and it is our goal to study methanogenic degradation of benzoate, it was necessary to create conditions favourable for methanogenesis to occur. Test experiments, incubated at 10 °C did not result in methanogenesis even after 200 days until the vials were transferred to 30 °C (2). For this reason, we decided to incubate the slurries prepared in this study at 30 °C, in the dark.

LC–MS method for benzoic acid measurements

LC–MS analysis was performed with a Vanquish UPLC system coupled to a Q Exactive Plus mass spectrometer (Thermo Fisher Scientific, Germany), using a heated electrospray ionization (HESI-II) source. Separation was performed on a C18 column (C18 BEH, 100 x 2 mm, 1,7 µm particle size, ACQUITY Waters, equipped with guard-column) with a flowrate of 0.45 mL/min (solvent A: H_2O + 0.1 % formic acid (FA), solvent B: acetonitrile (ACN) + 0.1 % FA). After injection, the samples were eluted isocratically at 8 % B for 4 min, followed by a gradient to 99 % B over 3.5 min and held for 0.7 min. The re-equilibration phase at 8 % B was 2.5 min. MS spectra were acquired in full MS mode with a resolution of 70,000 in negative mode and a scan range of 70 to 1050 m/z. The auxiliary gas and transfer capillary temperature was set to 350 °C, the spray voltage was 2.9 kV, the sheath gas flow rate was 45 and auxiliary gas rate 15. In order to ascertain high mass accuracy at the low mass range (70–1050 m/z), phenylacetic acid ($C_8H_8O_2$) and pyruvic acid ($C_3H_4O_3$) were added as calibrants at

20 µg per mL to the Negative Ion Calibration Solution (Pierce, Thermo Fisher). To limit the accumulation of salt deposits, the first 0.7 min of each run were discarded to waste. Each pore water sample was diluted 1:20 with water containing the injection standard (phenylacetic acid at a final concentration of 20 µg per mL). Benzoic acid was quantified relatively to the injection standard and an external calibration curve.

Metagenomic sequence data analysis

The resulting sequences (Table S5) were analysed with the MetaWRAP pipeline (3). First, sequencing adapters were removed and sequences were quality trimmed using the READ_QC module, a wrapper for that consistently applies quality and adapter trimming to FastQ files. Afterwards, the sequences were assembled with metaSPAdes assembly module (4). For binning of the scaffolds three different binning-programs, MaxBin2 (5), MetaBAT (6) and CONCOCT (7) were used. Bins refinement were then performed by bins hybridization from these three binning methods and bin selection based on CheckM estimation (8). To improve the bin quality, clean reads mapped to the each refined bins were reassembled individually. Furthermore, Prodigal software was used for gene prediction of reassembled bins by using the “meta” mode (9). For annotation, eggNOG-mapper, Interproscan and MMseqs2 vs. Uniclust database were used to annotate protein-coding regions (10-13).

Supplementary figures and tables

Table S1 Differences in crystallinity conductivity and half-life to reductive dissolution of the model iron oxides used in this study.

Iron oxide	Crystallinity	Conductivity	Half-life to reductive dissolution (days)	Reference
Hematite	Crystalline	Semi-conductive	182	
Magnetite	Crystalline	Conductive	72	(14)
Lepidocrocite	Poorly crystalline	Non-conductive	0.45	

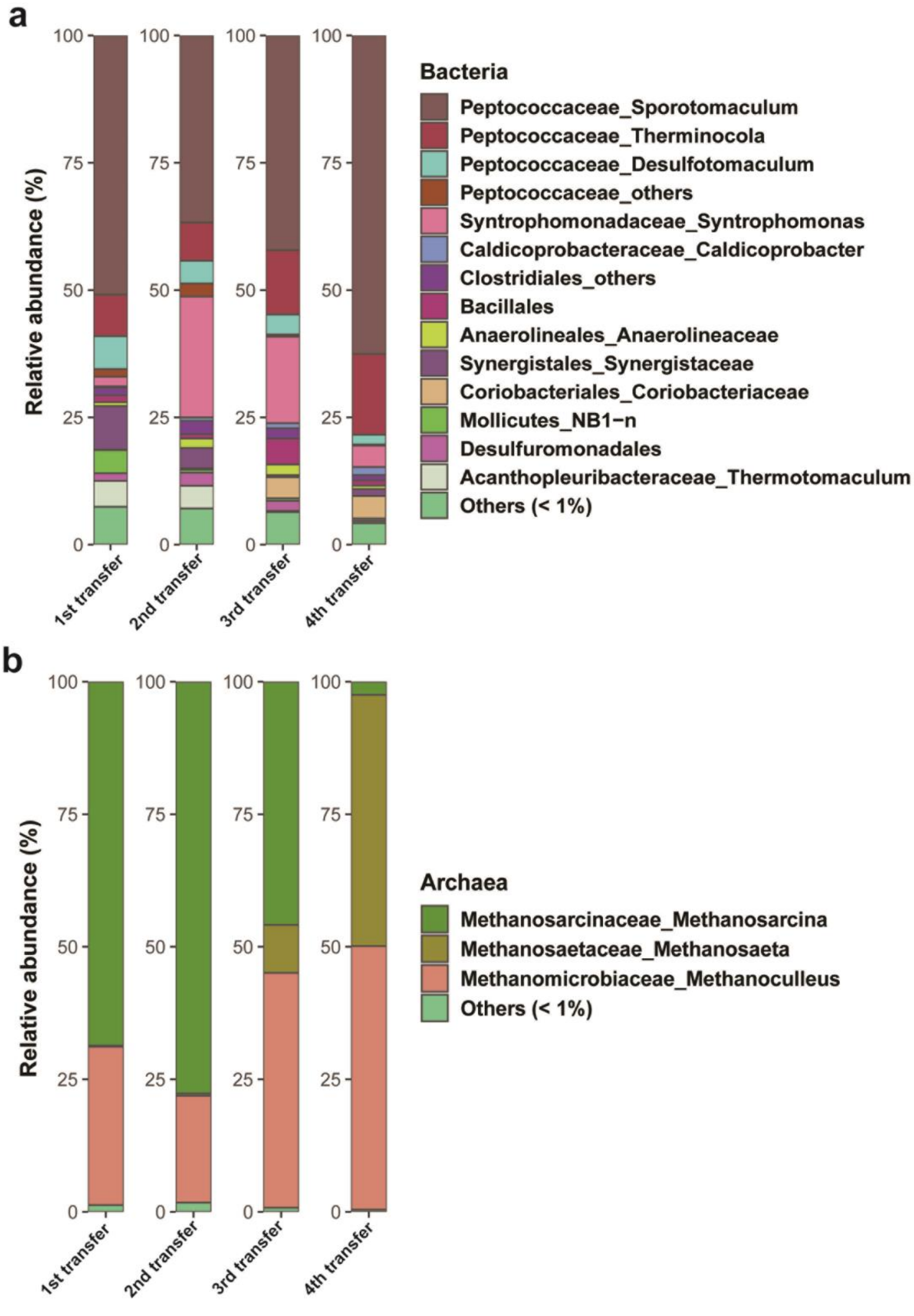

Fig. S1 Microbial community composition after four generation of transfers from the benzoate–magnetite sediment incubations. **a** Bacteria 16S rRNA gene sequences. **b** Archaea 16S rRNA gene sequences. Taxa are presented on order, family or class level for bacteria and on genus level for archaea.

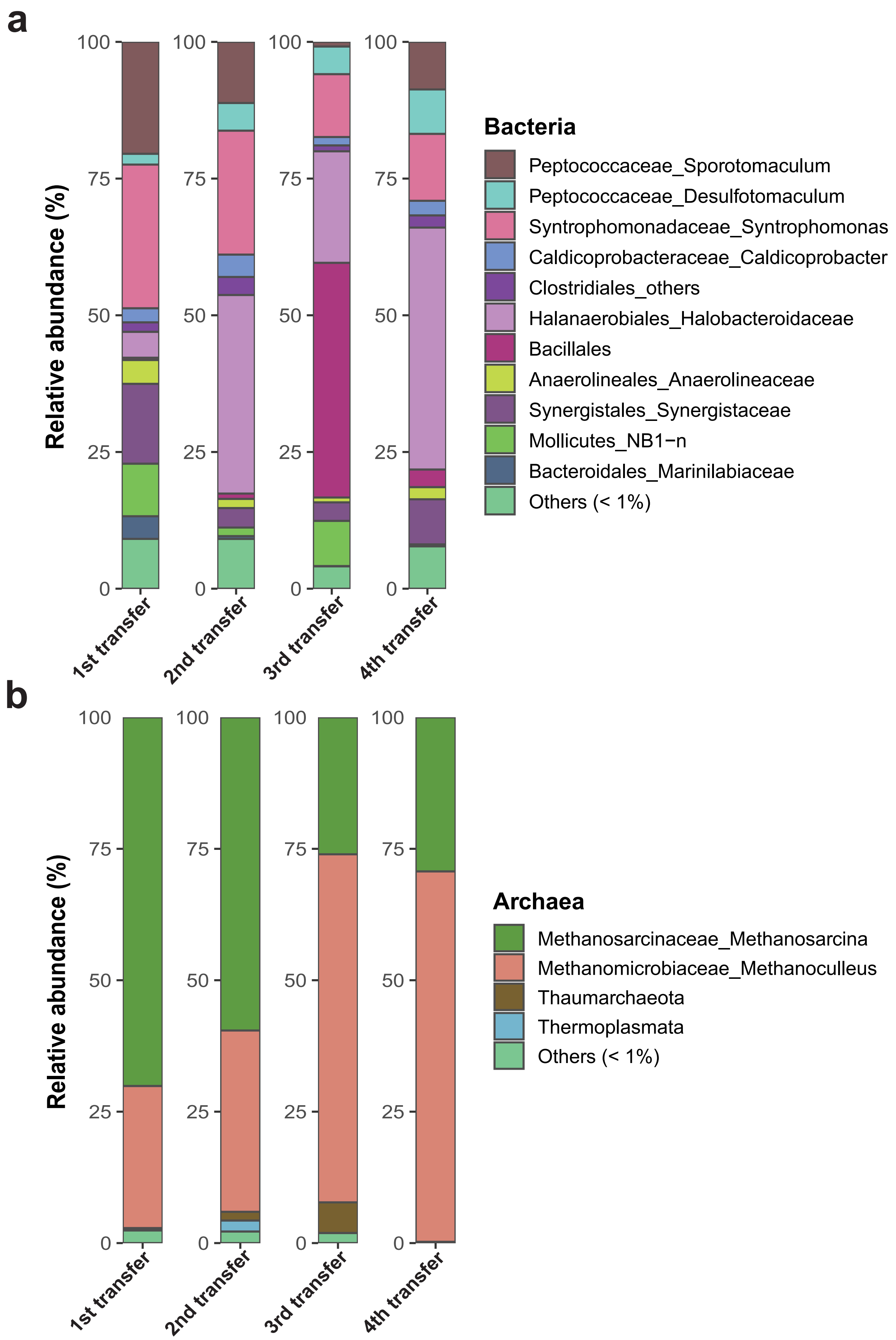

Fig. S2 Microbial community composition after 4 generation of transfers from the benzoate only sediment incubations. **a** Bacteria 16S rRNA gene sequences. **b** Archaea 16S rRNA gene sequences. Taxa are presented on order, family or class level for bacteria and on genus level for archaea.

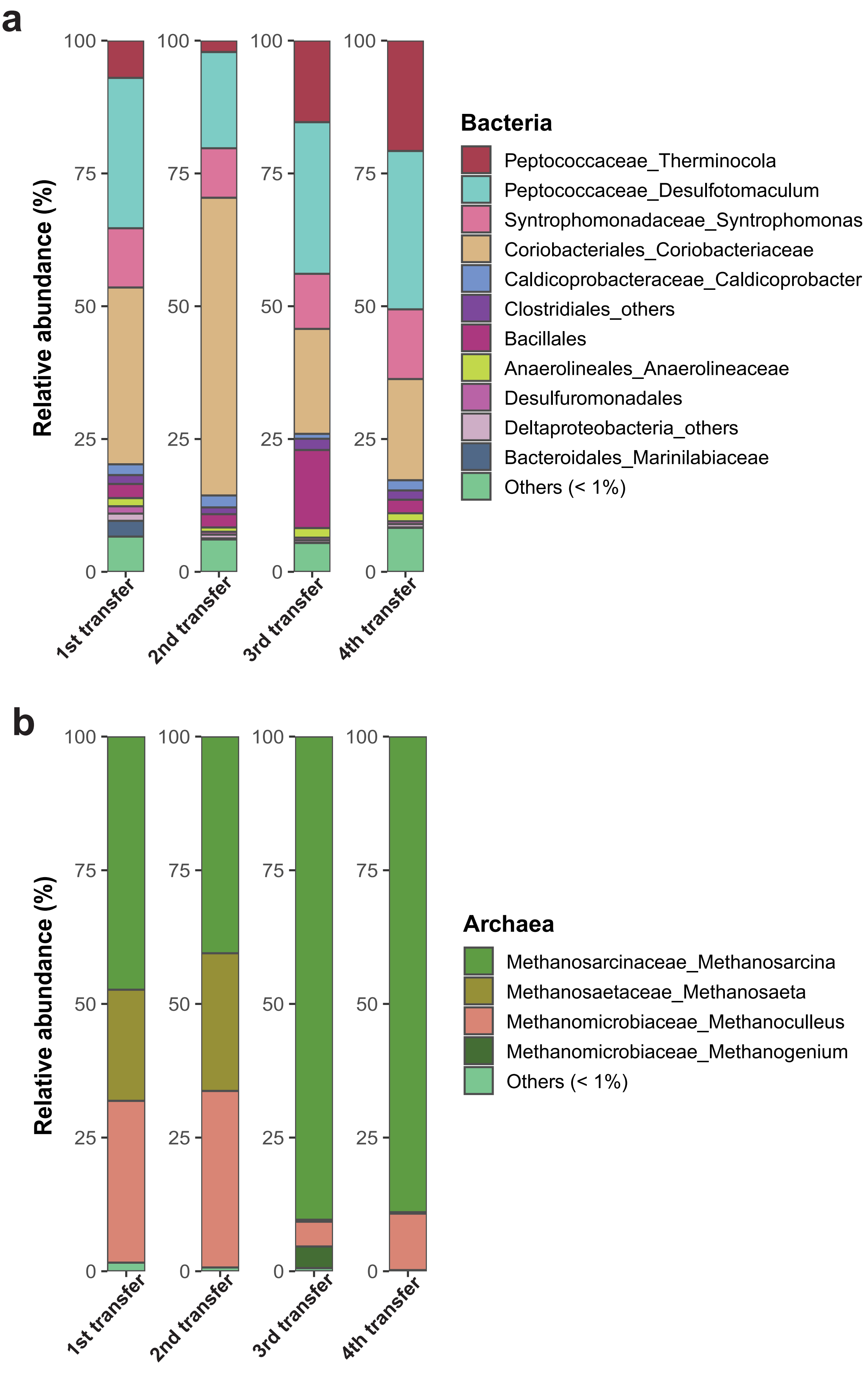

Fig. S3 Microbial community composition after four generation of transfers from the benzoate-lepidocrocite sediment incubations. **a** Bacteria 16S rRNA gene sequences. **b** Archaea 16S rRNA gene sequences. Taxa are presented on order, family or class level for bacteria and on genus level for archaea.

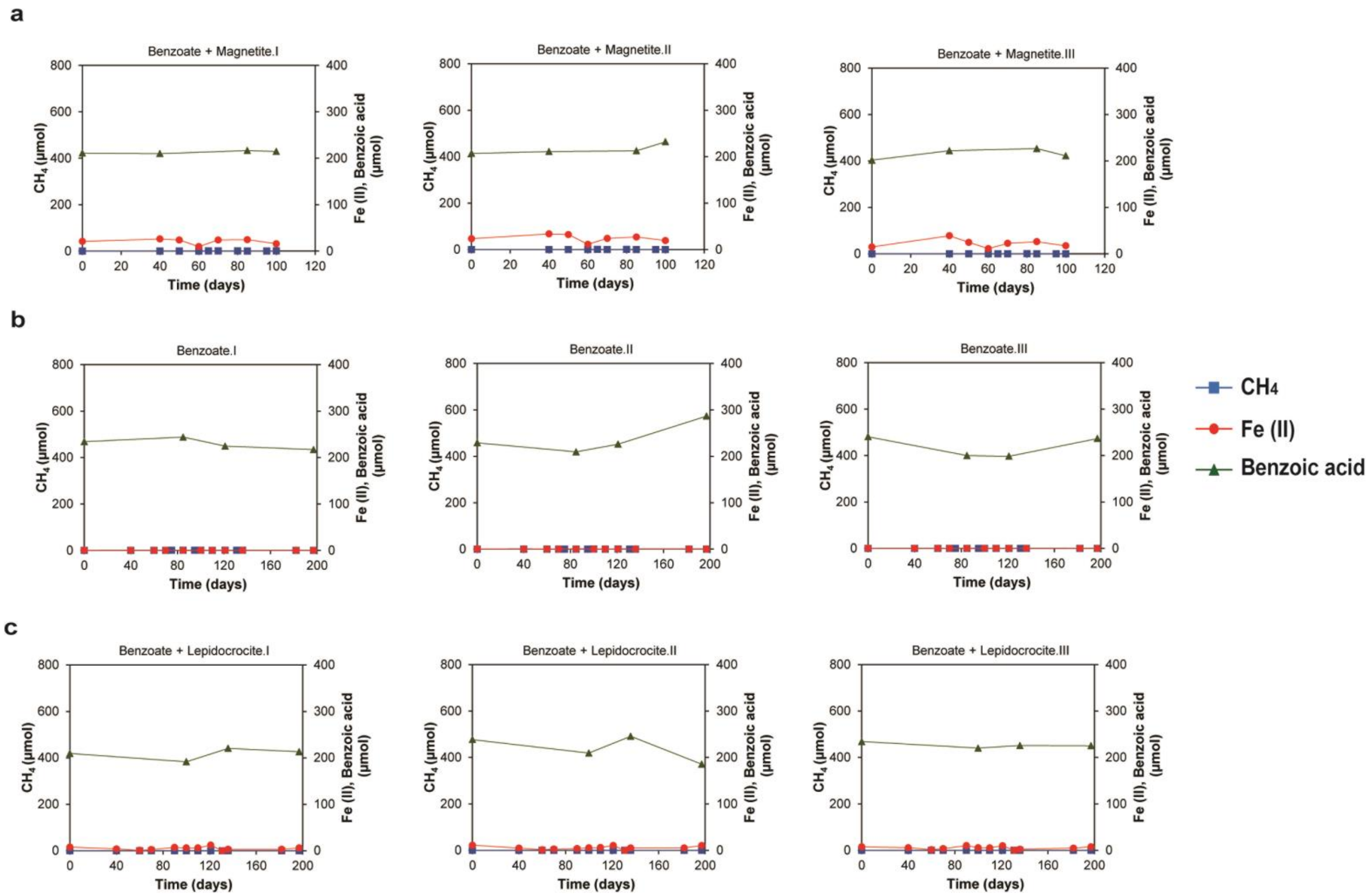

Fig S4 Kinetics of benzoate, methane and Fe(II) in control incubations without microbial cells from the 5th generation experiment. Butyrate and acetate were not detected. The absence of evidence for increased amounts of products or loss of benzoates confirm the processes observed in Fig. 2-4 of main article results from a biotic process.

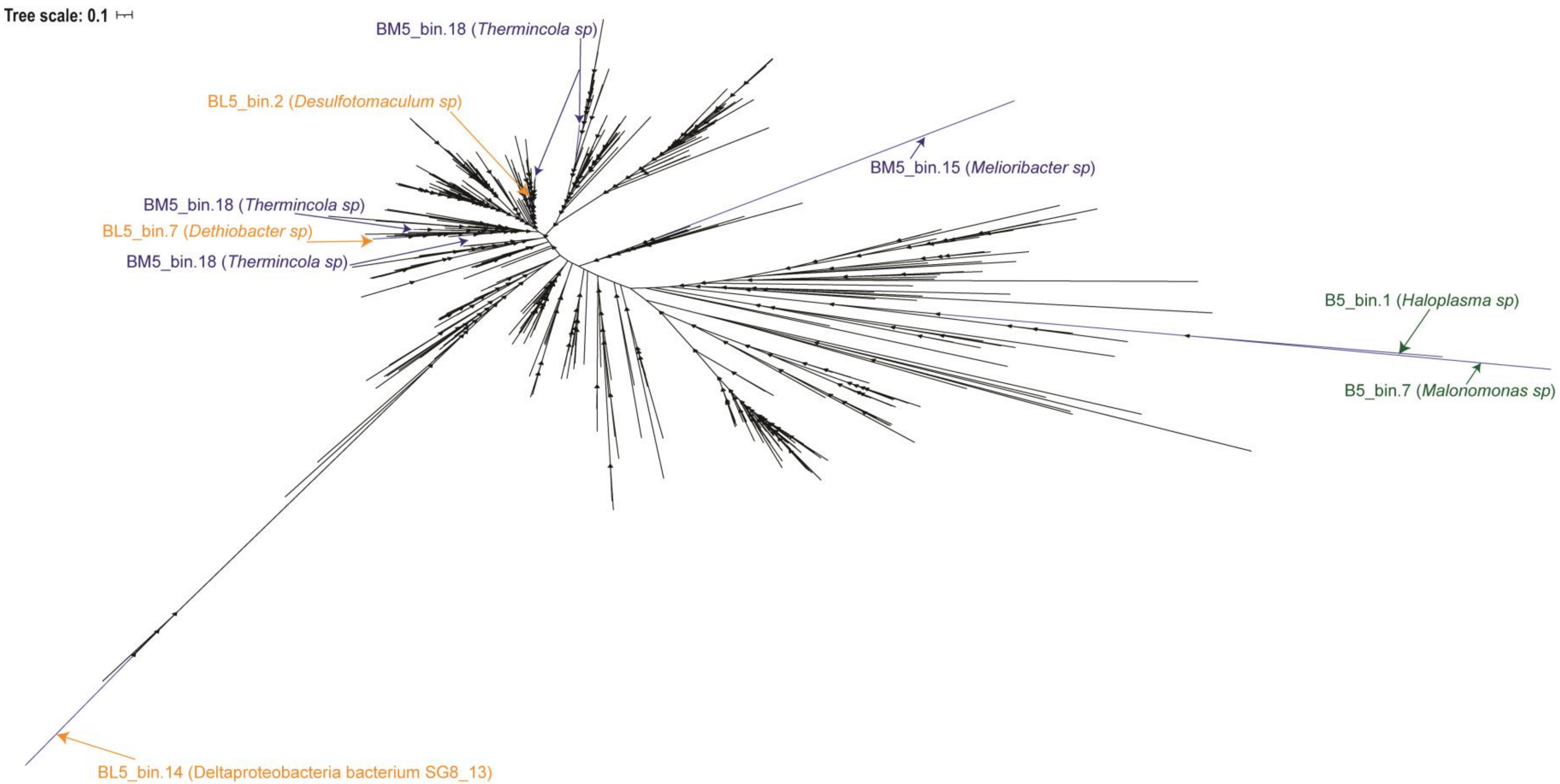

Fig S5 Genome assembled benzoate-CoA ligase genes tree including benzoate-CoA ligase genes found in the enrichments after 5 successive transfers.

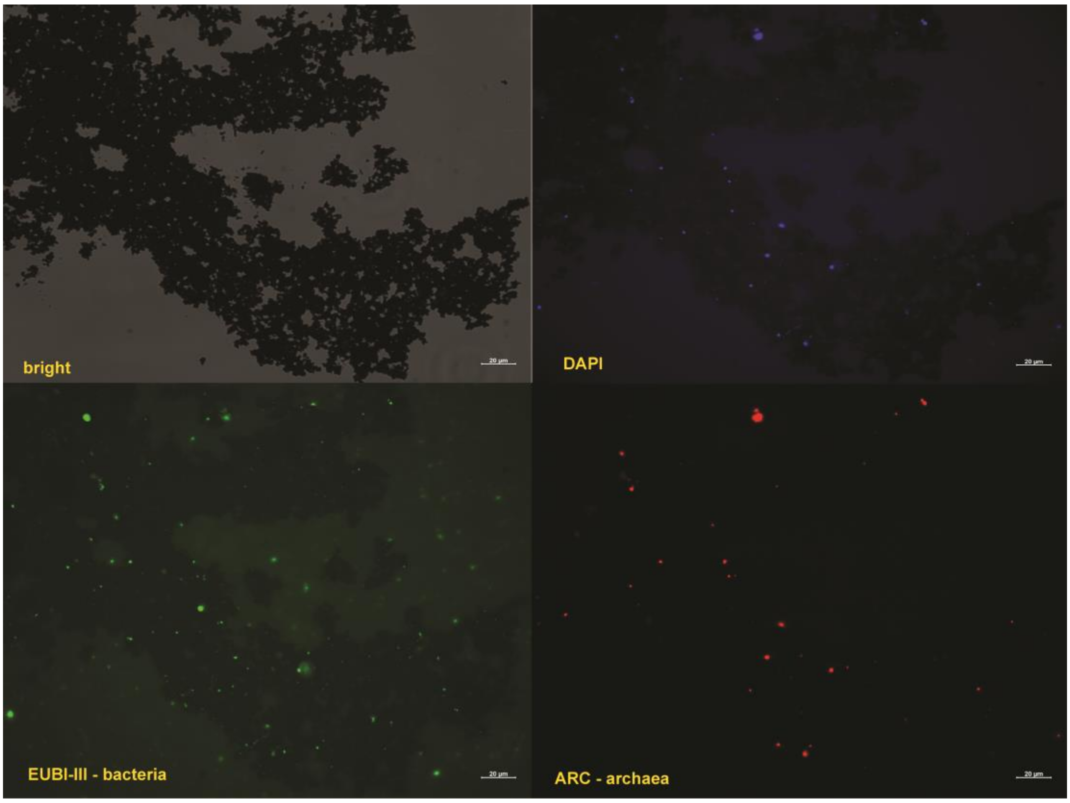

Fig. S6 CARD-FISH image of BM5 enrichment on various viewing channels.

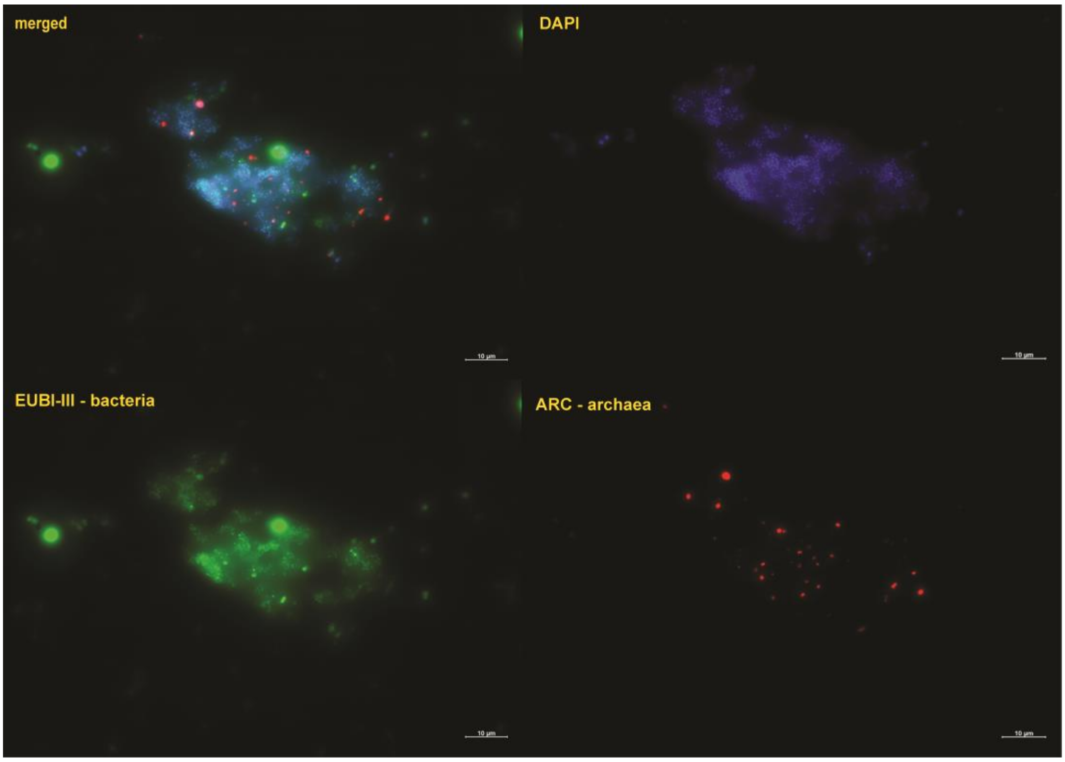

Fig. S7 CARD-FISH image of B5 enrichment on various viewing channels.

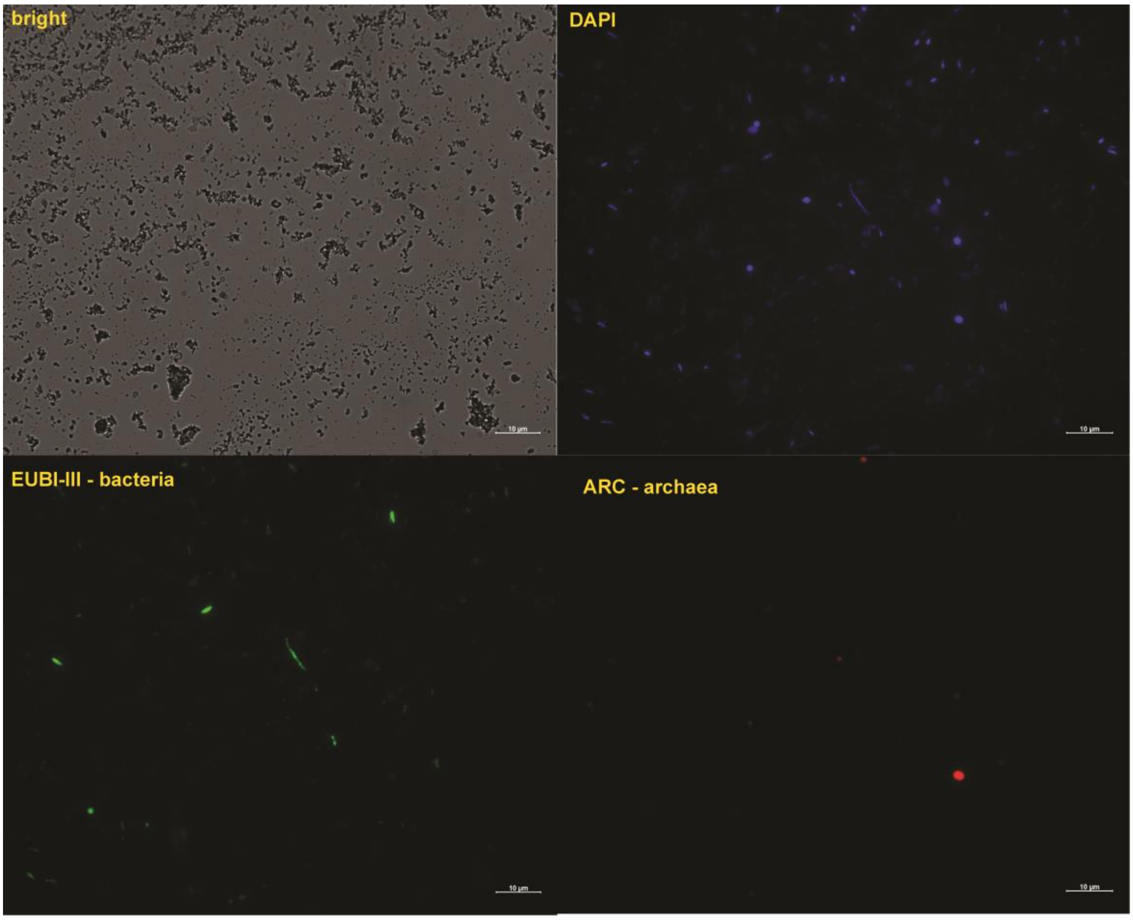

Fig. S8 CARD-FISH image of BL5 enrichment on various viewing channels.

Table S2 Quality metrics of the metagenomic bins containing genes encoding the various steps in the benzoate degradation pathway from the BM5 enrichment.

BM5 MAGs	Completeness (%)	Contamination	GC Content	N50	Size	Coverage	Contigs	Paired Reads (2x 300Bp)
bin.1	90.5	2.73	0.45	16930	2894654	7	197	170991
bin.10	98.8	1.59	0.44	165997	3382841	220	36	18280
bin.11	98	0	0.37	92960	3562057	8	88	23939
bin.12	57.8	0.67	0.33	2301	1337031	2.5	622	4391
bin.13	93.5	0	0.45	25790	2750548	12	143	83234
bin.15	71.4	1.49	0.36	6840	4074501	4	740	13949
bin.16	76.5	4.91	0.45	4224	2976530	3.5	780	7883
bin.18	63.5	5.27	0.42	89471	4156954	8	75	22717
bin.19	93.3	0	0.44	38721	2842865	9	81	7615
bin.2	98.7	2.91	0.49	189548	2855454	30	19	8381
bin.4	95.6	0.55	0.47	106554	3426057	8	49	19494
bin.5	93.2	4.69	0.37	12436	4547570	5	484	27626
bin.6	97.4	0	0.46	42539	3653553	260	67	71624
bin.7	90.9	0.65	0.62	102102	2432528	145	24	17677
bin.8	98.3	1.87	0.42	99571	3912647	76	56	10813
bin.9	87.2	2.94	0.43	24403	3276692	7	179	446991

Table S3 Quality metrics of the metagenomic bins containing genes encoding the various steps in the benzoate degradation pathway from the B5 enrichment.

B5 MAGs	Completeness (%)	Contamination	GC Content	N50	Size	Coverage	Contigs	Paired Reads (2x 300Bp)
bin.1	97.7	4.6	0.3	86229	3430657	9	73	62168
bin.11	67.9	4.8	0.32	5766	3609375	4	686	28142
bin.12	99.33	0.99	0.47	64219	4270005	39	91	271211
bin.13	95.7	0	0.44	230807	3042721	27	15	138831
bin.2	97.7	0	0.46	96929	2623609	11	55	52771
bin.4	91.2	0.96	0.45	55843	3083413	10	91	57048
bin.5	98.2	2.2	0.43	97363	3973561	52	90	351156
bin.6	43.2	1.5	0.43	3742	2083497	4	616	14100
bin.7	67.2	3.1	0.51	4808	3133879	3	742	24858
bin.8	54.2	1.2	0.52	3136	2641100	2.5	883	14391
bin.9	98.5	2.5	0.38	250195	5083581	85	43	725651

Table S4 Quality metrics of the metagenomic bins containing genes encoding the various steps in the benzoate degradation pathway from the BL5 enrichment.

BL5 MAGs	Completeness (%)	Contamination	GC Content	N50	Size	Coverage	Contigs	Paired Reads (2x 300Bp)
bin.1	98.8	0.38	0.46	150788	3880704	58	40	372334
bin.10	44.8	0	0.43	2899	2276154	3	807	15269
bin.11	93.1	2.91	0.45	522498	3204169	52	9	283187
bin.13	48.2	9.01	0.57	1576	2926831	2	1829	23663
bin.14	91.4	0.97	0.56	9980	5040300	7	687	50871
bin.16	93.1	5.64	0.46	1803718	3345903	19	8	107496
bin.17	98.2	3.23	0.43	179469	3583623	84	27	517206
bin.19	42.4	1.2	0.38	1651	1615649	2.5	978	8549
bin.2	98.6	3.18	0.47	34814	3844584	14	162	92303
bin.3	98.3	1.13	0.6	35238	3794241	40	160	217566
bin.4	98.8	0.64	0.45	110545	2838791	88	54	419013
bin.7	96.8	2.78	0.44	153211	4065565	23	36	157470
bin.8	92.5	2.08	0.35	10654	3009856	5	356	26068
bin.9	96.4	0	0.45	40683	2886690	21	91	92114

Table S5 Total number of paired reads in each enrichment.

	B5	BL5	BM5
Reads paired (2x 300Bp) raw	6844877	6281456	6684988
Reads paired (2x 300Bp) after trimming	6821245	6244155	6656244
metaspades contigs >=1kb	12831	13718	17080
Total length >= 1kb	69268701	74770412	83246197

References

1. Oni OE, Miyatake T, Kasten S, Richter-Heitmann T, Fischer D, Wagenknecht L, et al. Distinct microbial populations are tightly linked to the profile of dissolved iron in the methanic sediments of the Helgoland Mud Area, North Sea. Front Microbiol. 2015;6:365.

2. Oni OE. Structure and function of microorganisms in the methanic sediments of the Helgoland mud area, North Sea, Germany [PhD Thesis]. Bremen, Germany: University of Bremen; 2015.

3. Uritskiy GV, DiRuggiero J, Taylor J. MetaWRAP—a flexible pipeline for genome-resolved metagenomic data analysis. Microbiome. 2018;6(1):1-13.

4. Nurk S, Meleshko D, Korobeynikov A, Pevzner PA. metaSPAdes: a new versatile metagenomic assembler. Genome Res. 2017;27:824-34.

5. Wu Y-W, Tang Y-H, Tringe SG, Simmons BA, Singer SW. MaxBin: an automated binning method to recover individual genomes from metagenomes using an expectation-maximization algorithm. Microbiome. 2014;2(1):26.

6. Kang DD, Froula J, Egan R, Wang Z. MetaBAT, an efficient tool for accurately reconstructing single genomes from complex microbial communities. PeerJ. 2015;3:e1165.

7. Alneberg J, Bjarnason BS, De Bruijn I, Schirmer M, Quick J, Ijaz UZ, et al. Binning metagenomic contigs by coverage and composition. Nat Meth. 2014;11(11):1144-6.

8. Parks DH, Imelfort M, Skennerton CT, Hugenholtz P, Tyson GW. CheckM: assessing the quality of microbial genomes recovered from isolates, single cells, and metagenomes. Genome Res. 2015;25:1043-55.

9. Hyatt D, Chen G-L, LoCascio PF, Land ML, Larimer FW, Hauser LJ. Prodigal: prokaryotic gene recognition and translation initiation site identification. BMC Bioinformatics. 2010;11(1):119.

10. Huerta-Cepas J, Forslund K, Coelho LP, Szklarczyk D, Jensen LJ, Von Mering C, et al. Fast genome-wide functional annotation through orthology assignment by eggNOG-mapper. Mol Biol Evol. 2017;34(8):2115-22.

11. Jones P, Binns D, Chang H-Y, Fraser M, Li W, McAnulla C, et al. InterProScan 5: genome-scale protein function classification. Bioinformatics. 2014;30(9):1236-40.

12. Steinegger M, Söding J. MMseqs2 enables sensitive protein sequence searching for the analysis of massive data sets. Nat Biotechnol. 2017;35(11):1026-8.

13. Mirdita M, von den Driesch L, Galiez C, Martin MJ, Söding J, Steinegger M. Uniclust databases of clustered and deeply annotated protein sequences and alignments. Nucleic Acids Res. 2017;45(D1):D170-D6.

14. Poulton SW, Krom MD, Raiswell R. A revised scheme for the reactivity of iron (oxyhydr)oxide minerals towards dissolved sulfide. Geochim Cosmochim Acta. 2004;68(18):3703-15.
